# Impact of a Single Nucleotide Polymorphism on the 3D Protein Structure and Ubiquitination Activity of E3 Ubiquitin Ligase Arkadia

**DOI:** 10.3389/fmolb.2022.844129

**Published:** 2022-02-23

**Authors:** Maria Birkou, Vasilios Raptis, Konstantinos D. Marousis, Athanasios Tsevis, Kyriakos Bourikas, Detlef Bentrop, Vasso Episkopou, Georgios A. Spyroulias

**Affiliations:** ^1^ Department of Pharmacy, University of Patras, Patras, Greece; ^2^ School of Science and Technology, Hellenic Open University, Patras, Greece; ^3^ Institute of Physiology II, Faculty of Medicine, University of Freiburg, Freiburg, Germany; ^4^ Faculty of Medicine, Imperial College London, Hammersmith Hospital Campus, Burlington Danes, London, United Kingdom

**Keywords:** Arkadia, snps, NMR, RING, E3 ligase

## Abstract

Single nucleotide polymorphisms (SNPs) are genetic variations which can play a vital role in the study of human health. SNP studies are often used to identify point mutations that are associated with diseases. Arkadia (RNF111) is an E3 ubiquitin ligase that enhances transforming growth factor-beta (TGF-*β*) signaling by targeting negative regulators for degradation. Dysregulation of the TGF-*β* pathway is implicated in cancer because it exhibits tumor suppressive activity in normal cells while in tumor cells it promotes invasiveness and metastasis. Τhe SNP CGT > TGT generated an amino-acid (aa) substitution of Arginine 957 to Cysteine on the enzymatic RING domain of Arkadia. This was more prevalent in a tumor than in a normal tissue sample of a patient with colorectal cancer. This prompted us to investigate the effect of this mutation in the structure and activity of Arkadia RING. We used nuclear magnetic resonance (NMR) to analyze at an atomic-level the structural and dynamic properties of the R957C Arkadia RING domain, while ubiquitination and luciferase assays provided information about its enzymatic functionality. Our study showed that the R957C mutation changed the electrostatic properties of the RING domain however, without significant effects on the structure of its core region. However, the functional studies revealed that the R957C Arkadia exhibits significantly increased enzymatic activity supporting literature data that Arkadia within tumor cells promotes aggressive and metastatic behavior.

## Introduction

Ubiquitination is a three-step enzymatic reaction catalyzed by the action of three key-enzymes. Initially, ubiquitin is activated in an ATP-dependent manner by the E1 activating enzyme. Activated ubiquitin afterwards is transferred to E2 conjugating enzyme. In the last step of the enzymatic reaction ubiquitin is covalently attached to the target protein, through an isopeptide bond between its G76 and the ε-amino group of a lysine residue of the target protein. The attachment of ubiquitin to substrate is catalyzed by the E3 ubiquitin ligase. The E3 ubiquitin ligases are considered crucial partners of the ubiquitination machinery because they act as specific substrate recognition elements. The ubiquitin ligases are characterized by the presence of either a HECT (Homologous to E6-AP Carboxyl Terminus) or a RING (Really Interesting New Gene) domain (Hershkos et al., 1983; Pickart, 2001). RING-type E3 ligases constitutes the largest class of E3 ligases and they are characterized by the following amino acids sequence: Cys-X_2_-Cys-X_9-39_-Cys-X_1-3_-His-X_2_-Cys/His-X_2_-Cys-X_4-48_-Cys-X_2_-Cys, wherein X represents any amino acid. The RING domain motif is constructed by Cys2His2, Cys3His or Cys4 metal binding sites which coordinate two zinc ions in a “cross-brace” arrangement (Freemont, 1993; Saurin et al., 1996).

Ubiquitination is a signaling pathway that regulates a broad spectrum of biochemical processes, e.g., it enhances the tumor suppression activity of some proteins or the oncogenic and metastatic properties of others and has a significant role in the life cycle of cells. Thus, many studies have shown that mutations in E3 ligases are implicated in cancer (Shangary and Wang, 2008; Nelson and Holt, 2010; Sharma et al., 2011; Chan et al., 2013; Wang et al, 2017) and other diseases (Kumar et al., 2017). Two of the thoroughly studied and well characterized RING E3 ligases are BRCA1 and HDM2. Tumor suppressor BRCA1 is a RING E3 ligase whose mutations lead to a high predisposition of breast and ovarian cancers (Brzovic et al., 2001; Rosen, 2013). Mutations observed in the RING sequence are now considered as significant markers for these types of cancer. Similarly, Hdm2 is a RING E3 ligase whose overexpression associates with many human tumor types (breast, esophageal, lung carcinomas etc.,), whereas its binding and degradation of p53 tumor suppressor is inactivated in more than 50% of human cancers (Moll and Petrenko, 2003; Sun, 2006). Targeting specific E3 ligases, that play a significant role in cancer, may contribute to the development of therapeutic strategies (Lakshmanan et al., 2008).

Arkadia (RNF111) is a RING ubiquitin ligase that positively regulates the TGF-*β* pathway by targeting its negative regulator SKI and its close homologue SKIL (SNON), as well as the inhibitory SMAD7 for ubiquitin-dependent proteasomal degradation. Arkadia functions as E3 ligase through its C-terminal RING-H2 domain (amino acids 942–983) (Levy et al., 2007; Mavrakis et al., 2007; Nagano et al., 2007). Arkadia was shown to enhance and support TGF-*β* tumor suppressing function in colorectal cancer (CRC) (Sharma et al., 2011). Furthermore, Le Scolan, et al., 2008 and Briones-Orta et al., 2013, using tumor cell lines driven by the TGF-*β* pathway, demonstrated that Arkadia has a potent tumor-promoting activity. These data indicate that Arkadia supports both properties of the TGF-*β* pathway, i.e., tumor suppression in normal cells and metastasis in tumor cells. Moreover, deep sequencing studies of the mRNA from tumors of CRC patients (Bravou et al., 2009; Sharma et al., 2011), led to the identification of somatic mutations that diminish Arkadia’s function (Sharma et al., 2011). They were listed in the COSMIC database of somatic mutations in cancer (Bravou et al., 2009; Sharma et al., 2011).

In the present study the single nucleotide polymorphism (SNP) of arginine (R) 957 (rs780099637, CGT > TGT, allele frequency = 0.000004 in gnomAD) enriched in CRC was studied. SNPs are genetic variations that are associated with individual susceptibility to diseases (Shastry, 2007). The R957C SNP was identified both in CRC and adjacent normal tissue samples (Supplementary Table S1). Existence of mutations in normal tissues adjacent to tumors is frequently observed and their association with cancer is under investigation (Risques and Kennedy 2018; Fiala and Diamandis, 2020; Oh and Sung, 2020). SNP studies are crucial to identify amino acids substitutions; in the protein coding regions that potentially alter the function or structure of a protein (Bhattacharya et al., 2017). Herein, we present a thorough overview about the effects of the R957C point mutation. Replacement of the positively charged arginine with cysteine affects the electrostatic properties of the protein, which may lead to alterations of structure, stability, and function of the enzymatic RING domain of the E3 ubiquitin ligase Arkadia. To predict the effect of the R957C mutation on Arkadia protein, the SNPs and GO and I-Mutant pathogenicity prediction servers were used (Capriotti et al., 2005; Capriotti et al., 2013). In order to investigate the impact of this mutation on the conformational dynamics and the function of the Arkadia RING domain, we prepared the recombinant polypeptide bearing a cysteine residue instead of arginine in position 957 of the human sequence and we conducted a structural and functional study to obtain an atomic-level insight into its conformational dynamics and activity in comparison with the native Arkadia RING domain.

## Materials and Methods

### Prediction of Single Nucleotide Polymorphism Impact

The effect of the R957C substitution was analyzed using SNPs and GO and I-Mutant tools. SNPs and GO is a tool which predicts disease associated amino acid substitution at a single position in a specific protein including functional classifications with >82% prediction accuracy. I-Mutant predicts the effects of single point mutation with 80% accuracy. A probability score higher than 0.5 reveals a disease related effect of the mutation on protein function. The input given to the SNPs and GO and I-Mutant tools was the UniProt accession number (Q6ZNA4) of Arkadia isoform-1 protein, the sequence position of the wild type amino acid and the mutated amino acid.

### Protein Expression and Purification

A human RNF111 gene encoding amino acids 927–994 of the full length Arkadia was sub-cloned in a pGEX-4T-1 vector and transformed in *Escherichia coli* (*E. cloni*®) EXPRESS BL21 (DE3) cells (Lucigen). Cells were grown at 37°C in minimal medium (M9) supplemented with 1 g/L^15^N ammonium chloride, 4 g/L glucose or ^13^C-glucose and 1 ml/L^15^N or ^15^N/^13^C Bioxpress™ (CIL) for single or double labeled samples, respectively. Cell cultures were induced at an optical absorption of 0.6–0.9 with 1 mM IPTG (final concentration). After 4 h incubation at 37°C cells were harvested and the resulting pellet was lysed by sonication in PBS (Phosphate Buffered Saline) pH 7.4 ± 0.2 containing a protease inhibitor cocktail (Sigma) and DNAase I. The cell lysate was cleared by centrifugation at 20.000rpm (rounds per minute) for 30 min. For protein purification the supernatant was loaded onto a GST-trap 5 ml column (GE Healthcare). The GST-tag and Arkadia^927-994^ were separated after overnight incubation with the protease thrombin (Merck Millipore) at 4°C. Arkadia^927-994^ was eluted in PBS pH 7.4 ± 0.2 and concentrated using Amicon® Ultra centrifugal filter units (3 kDa cutoff) (Merck Millipore). The protein was further purified by size exclusion chromatography on a Superdex75 column (GE Healthcare).

The E2 enzyme UBCH5B (UBE2D2) was expressed and purified as described elsewhere (Birkou et al., 2017) and ubiquitin was expressed in *E. coli* (*E. cloni*®) EXPRESS BL21 (DE3) cells (Lucigen). Cells were grown at 37°C in minimal medium (M9) supplemented with 4 g/L glucose and 1 g/L NH_4_Cl and induced with 1 mM (final concentration) IPTG. After 4 h of incubation at 37°C cells were harvested and lysed by sonication in PBS pH 7.4. The cell lysate was cleared by centrifugation at 20.000 rpm for 30 min. The supernatant was heated at 85°C for 15 min and centrifuged at 10.000 rpm for 20 min. For further purification ubiquitin was passed through a Superdex75 column (GE Healthcare).

### Nuclear Magnetic Resonance Data and Structure Calculation

NMR spectra were recorded on a Bruker Avance 600 MHz spectrometer equipped with a TXI cryoprobe and Bruker Avance III 700 MHz spectrometer equipped with a four-channel 5 mm cryogenically cooled TCI gradient probe. Protein samples were prepared in a mixed solvent of 90% H_2_0 (50 mM K_2_HPO_4_, 50 mM KH_2_PO_4_ pH 7), 10% D_2_0, 1 mM NaN_3_ and 0.25 mM DSS (4,4-dimethyl-4-silapentane-1-sulfonic acid) as internal chemical shift standard. All NMR experiments are included in the Bruker pulse program library. Data were processed with Topspin 3.5 pl5 software and analyzed with CARA (Keller, 2004) and XEASY (Bartels et al., 1995). The sequence specific assignment of R957C Arkadia^927−994^ domain was obtained using conventional backbone and side chain assignment methods (Wüthrich, 1986; Ferentz and Wagner, 2000) and were deposited in the BioMagResBank (BMRB; accession no: 50,985). The NMR solution structure of the R957C mutant was determined with DYANA (Güntert et al., 1997) and the ensemble of 30 models with the lowest RMSD and target function values was deposited in the ProteinDataBank after energy minimization with AMBER (Pearlman et al., 1995) (PDB, accession no: 7P2K; statistical analysis is reported in Supplementary Table S2). The assignment of the backbone ^1^H-^15^N resonances of all non-proline residues of E2 enzyme UBCH5B was obtained from the BMRB database (accession no: 6,277).

### ^15^N Relaxation Data

The backbone mobility of the R957C Arkadia^927-994^ mutant on the ps-ns time scale was investigated through ^15^N relaxation measurements (^15^N *T*_1_ and *T*_2_, {^1^H^N^}-^15^N NOE at 298 K) on Bruker Avance 600 and Avance III 700 spectrometers (experiments are reported in Supplementary Table S3). Standard pulse programs of the Bruker library were used. The overall delays between scans were 1.8 s for the T_1_ and T_2_ measurements and 4 s for the interleaved heteronuclear NOE experiment, respectively. The delays used for the T_1_ experiments were 7, 18, 40, 85, 150, 230, 350, 500, 680, and 900 ms, whereas delays of 17, 34, 51, 68, 85, 102, 136, 187, and 221 ms were used for the T_2_ experiments. ^15^N relaxation data were analyzed according to the model-free approach as implemented in the Tensor 2 program (Dosset et al., 2000) (*R*_1_, *R*_2_ and (^1^H) ^15^N NOE diagrams are shown in Supplementary Figure S2).

### Atomic Absorption Spectroscopy

To identify whether two zinc ions are bound to the R957C mutant, atomic absorption spectroscopy was performed (Perkin Elmer AAnalyst 700). For the zinc concentration determination proteins were purified as described above. Zinc ions standards of 0.1–1 ppm (Zinc Pure Standard, 1000 μg/ml, 2% HNO_3_, Perkin Elmer) were used and an appropriate calibration curve was constructed (Supplementary Figure S1). The concentration of zinc in the protein samples was determined dividing the intercept by the slope of calibration curve.

### Metal-Chelation Experiments

To evaluate the stability of the R957C Arkadia^927–994^ domain metal-chelation experiments were performed with the ethylenediaminetetraacetic acid (EDTA). ^1^H-^15^N HSQC spectrums were recorded after each EDTA addition at a ratio 1:0.5, 1:1, 1:1.5 and 1:2 (Zn: EDTA).

### Titration Experiments Monitored by Nuclear Magnetic Resonance

In order to identify the interaction interface between E3 R957C Arkadia^927–994^ and E2 UBCH5B, titration experiments were monitored by ^1^H–^15^N HSQC spectra of labeled ^15^N R957C Arkadia^927–994^ or ^15^N UBCH5B after each addition of the unlabeled protein partner. The unlabeled protein was added in eight steps in order to reach the following ratios and saturation of labeled/unlabeled protein: 1:0.25, 1:0.5, 1:0.75, 1:1, 1:1.25, 1:1.5, 1:1.75, 1:2. Combined chemical shift perturbations (CSPs) after binding were calculated using the equation: $\Delta \delta_{ppm}=\sqrt{{\left(\Delta \delta_{HN}\right)}^{2}+{\left(\frac{\Delta \delta_{Ν}}{5}\right)}^{2}}$ (Garrett et al., 1997; Baker et al., 2006; Marousis et al., 2018).

### Ubiquitination Assay

For the ubiquitination assays the Arkadia^876–994^ polypeptide bearing the R957C mutation was used. Briefly, ubiquitination assay was performed by incubating 0.5 μM hUbe1 enzyme (Boston Biochem), 5 μM UBCH5B, 100–150 μM ubiquitin and 15 μM wt/R957C Arkadia^876-994^ in 20 mM Tris-HCl pH 7.5, 50 mM NaCl, 5 mM ATP, 2 mM MgCl_2_ and 2 mM DTT at 37°C for 0–60 min. Time points were collected after the addition of ATP and the reactions were stopped by addition of SDS loading buffer. Samples were resolved by 15% SDS-PAGE and visualized by western blotting for ubiquitin using the anti-Ub antibody P4D1 (Santa Cruz Biotechnologies, SCBT) and the goat anti-mouse m-IgGk BP-HRP sc-516102 (SCBT).

### Luciferase Assays

Luciferase assays were conducted in HEK293T cells (Cancer Research UK). using the Dual Luciferase reporter system (Promega), as previously described by Levy at al. 2007. Briefly, cells were transfected with the appropriate combination of promoter-reporter constructs and expression plasmids using FuGENE transfection reagent (Promega) and were cultured for 24 h after transfection. Luciferase activities in the cell lysates were measured following the manufacturer’s protocol. The experiments were repeated at least three times.

## Results

**FIGURE 1:**
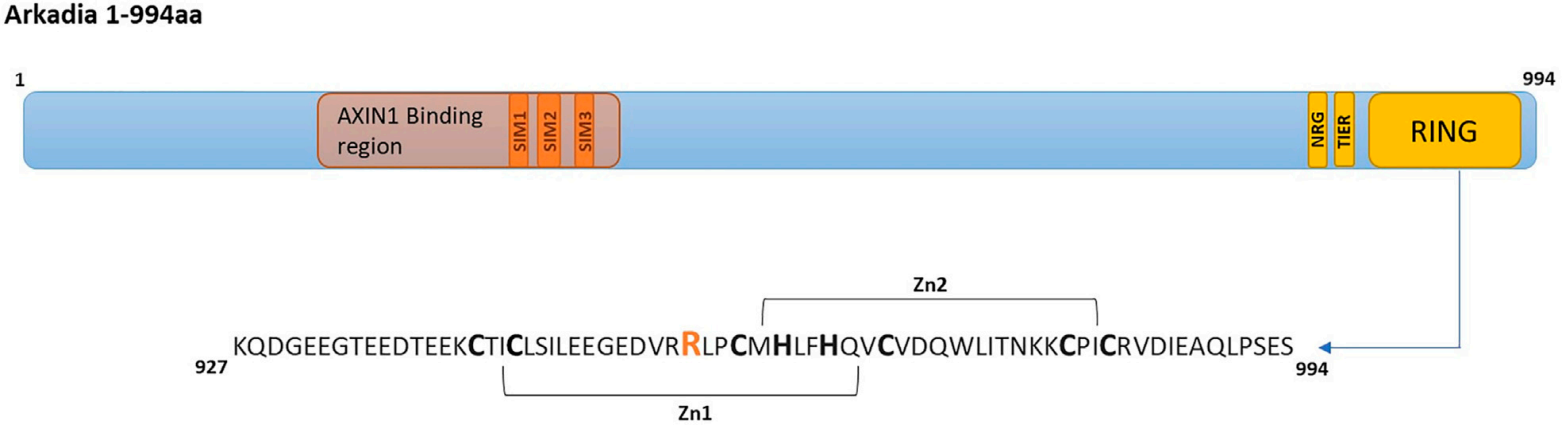
Arkadia E3 ubiquitin ligase domain organization and RING mutated sequence. SIMs (SUMO interacting motifs) (Poulsen et al., 2013) colored in orange, NRG, TIER segments and RING domain are colored in yellow.

The SNP and GO and I-Mutant pathogenicity prediction tools indicated, with high probability, that the R957C mutation in the Arkadia RING domain (Figure 1) is a disease-causing mutation (Supplementary Table S1). To determine the effect of the R957C mutation on Arkadia’s RING domain structure, stability and interaction properties NMR studies were performed. In addition, auto-ubiquitination and luciferase assays were carried out to investigate the biochemical activity of the R957C mutant.

### Determination of R957C Arkadia^927–994^ Zinc Ions Content and Stability Upon Addition of Ethylenediaminetetraacetic Acid.

Atomic absorption spectroscopy was performed on R957C mutant to measure the Zn (II) content. A protein sample of 0.3 mM gave zinc concentration of 0.71 mM. These concentrations correspond to two zinc ions per protein molecule.

**FIGURE 2:**
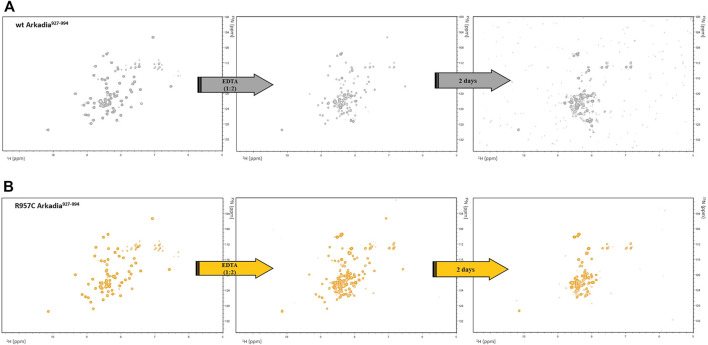
^1^H-^15^N HSQC spectra of **(A)** wt and **(B)** R957C RING before and after addition of EDTA.

According to the structural role of the Zn (II) in RING domains, addition of the metal-chelating agent EDTA to a final ratio 1:2 (Zn: EDTA) led to the complete unfolding of the protein after 48 h as indicated by the loss of chemical shift dispersion in the ^1^H-^15^N HSQC spectra (Figure 2A). Similarly, addition of EDTA to the wt RING domain caused complete unfolding of the protein after 48 h of incubation with the chelating agent (Figure 2B). These results suggest that the addition of one more Cys residue in the RING domain of Arkadia has not affected the zinc ions binding and the stability of the mutated protein. According to the C_α_ (56 ppm) and C_β_ (27 ppm) chemical shifts Cys957 in reduced state and does not participate in zinc ions binding or in disulfide bond formations (Sharma and Rajarathnam, 2000).

### Solution Structure and ^15^N Relaxation Studies of the R957C Arkadia^927–994^ RING Domain

**FIGURE 3:**
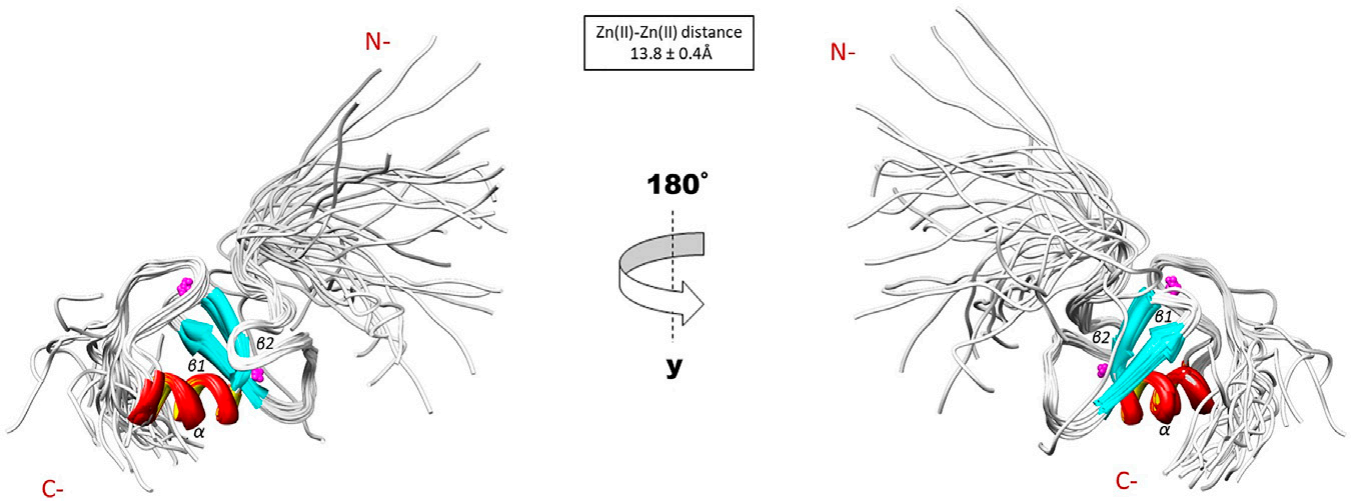
Solution structure of the R957C RING domain as represented by the final family of 30 energy minimized models.

**FIGURE 4:**
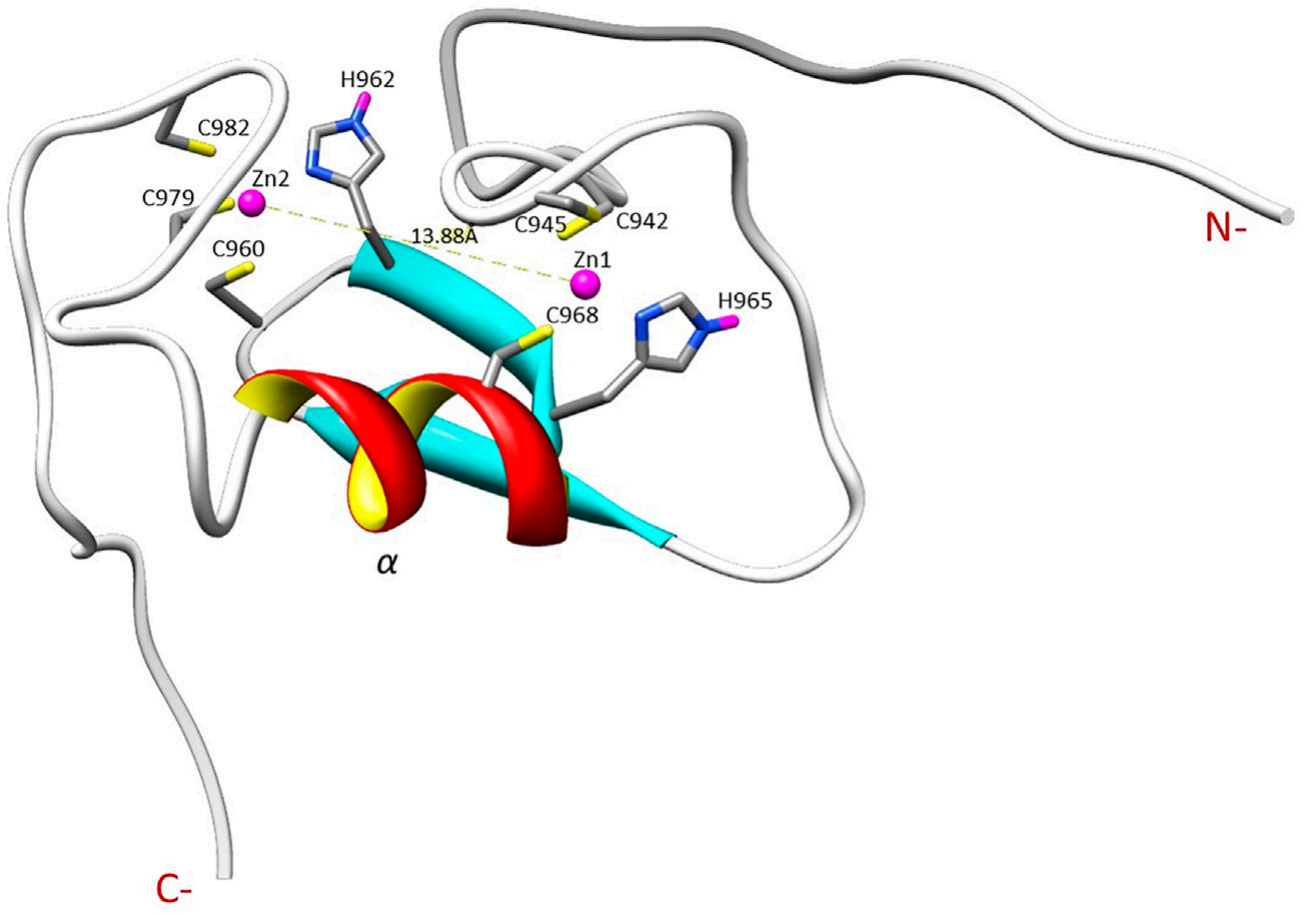
Illustration of the zinc ions binding sites and the Zn ions distance in the R957C RING domain as appeared in the first model of the family.

The NMR solution structure of the R957C mutant was determined based on a total of 906 NOEs distance constraints. The final family energy minimized NMR models contains two *β*-strands, namely *β*1 (Val955-Leu958) and *β*2 (His962-His965) forming an antiparallel *β*-sheet, two zinc binding loops and a 3-turn *α*-helix encompassing the residues Gln966-Thr975 (Figure 3). The two *β*-strands of the R957C mutant are one amino acid longer compared to wt RING Val955-Arg957 and Leu963-His965 *β*-strands. The *a-*helix observed in the R957C mutant is identical to the wt Arkadia RING helix in length and sequence position (Chasapis et al., 2012). The secondary structure elements of the R957C RING domain exhibit a *ββα* topology as observed in the wt Arkadia, RNF24 (PDB: 2EP4) and RNF168 RING domains (Zhang et al., 2013). The average distance between the two metal centers in the final 30 NMR models is 13.8 ± 0.4 Å (Figure 3). A detailed view of the coordination of the two Zn (II) ions is shown in Figure 4. The overall backbone and heavy atom RMSDs of the 30 energy-minimized models of R957C are 1.22 ± 0.48 Å and 2.08 ± 0.45 Å, respectively, for the core region between Glu939 and Ile986. Quality assessment of the final models reveals that 100% of the non-glycine/non-proline residues fall into favorable or allowed regions of the φ/ψ dihedral angle space in the Ramachandran plot (Supplementary Table S2).

**FIGURE 5:**
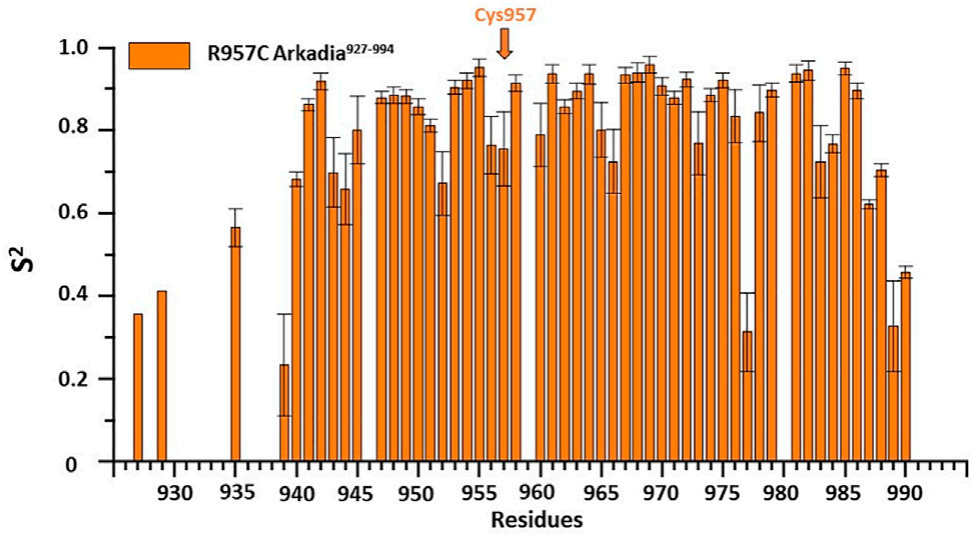
Order parameters (*S* ^*2*^) derived from ^15^N-relaxation measurements [*R*_*1*_, *R*_*2*_ and heteronuclear (^1^H^N^)– ^15^N NOE] for R957C Arkadia^927–994^ domain at 600 MHz and 298 K.

^15^N relaxation studies of R957C Arkadia^927–994^ revealed that mutant is a monomer in solution since the correlation time for isotropic tumbling measured based on the *R*_2_/*R*_1_ ratio is 4.69 ns corresponding to a MW of ∼7.81 kDa (theoretical MW of R957C RING is 7767 Da). In addition, model-free analysis of ^15^N relaxation data as implemented in the Tensor2 program showed that the core exhibited a rather rigid structure (Figure 5). The order parameters of the region Lys941-Ile986 (average *S* ^2^ = 0.77) are higher than those of the N- and C-terminal residues (Lys927-Glu940 and Glu987-Ser994, respectively) closely resembling the ^15^N-relaxation properties of the wt Arkadia RING (Chasapis et al., 2012; Birkou et al., 2017) (Figure 5). The above data clearly support that there are no significant differences in the structure and dynamic properties between wt and R957C Arkadia RING domain.

### Interaction Studies of R957C Arkadia^927–994^ Really Interesting New Gene Domain and UBCH5B

**FIGURE 6:**
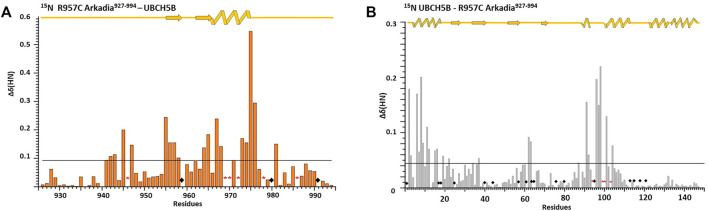
Interaction of E3 Ub ligase Arkadia RING with the E2 UBCH5B enzyme monitored by ^1^H-^15^N HSQC spectra. Diagram **(A)** illustrates the total CSPs measured at 1:2 M ratio for ^15^N R957C Arkadia/^14^N UBCH5B (left), while diagram **(B)** illustrates the CSPs for ^15^N UBCH5B/^14^N R957C Arkadia RING. Straight lines indicate the applied thresholds of CSPs representing the respective average CSP (Kornhaber et al., 2006). ^◆^represents proline residues and residues with no information.

To determine whether the R957C mutation affects the interaction of the RING with E2 we used NMR driven interaction studies of ^15^N R957C with the E2 enzyme UBCH5B, which functions as a partner of Arkadia *in vitro* (Mavrakis et al., 2007). The analysis showed that the resonances exhibit either fast or intermediate exchange behavior, suggesting a moderate affinity for the two proteins. The largest chemical shift changes were observed for the sequential stretches Cys942–Thr943, Cys945-Ile947, Val955-Leu958, Phe964-His965, Val967-Asp970 and Trp972-Asn976 and for residue Ile981 (Figure 6A). The first three sequential stretches comprise the first and the third Zn(II)-binding motif, while the fourth and fifth are located at the C-end of the *α*-helix Gln966-Thr975. The above regions are essentially identical with those exhibiting the largest chemical shift changes upon interaction with UBCH5B in the native RING (Chasapis et al., 2012; Birkou et al., 2017).

The addition of unlabeled R957C RING to ^15^N-labeled UBCH5B resulted in the loss of four UBCH5B amide resonances (Ser94, Ile99, Ser100 and Leu103) in the ^1^H–^15^N HSQC spectra during the titration. The largest CSPs in UBCH5B were observed for residues Ala2-Leu3, Ile6-Glu9, Asn11-Asp12, Arg15-Asp16 (N-terminal helix *α*1) Ala19, Ser22 (*β*1 strand), Ile37 (*β*2 strand), Phe62-Lys63 (loop L1 between the *β*3 and *β*4 strands), Ser91-Glu92, Ala96-Thr98 (loop L2b), Lys101, and Leu104 (the first half of helix *α*2) (Figure 6B). All these residues were also found to participate in wt Arkadia RING–UBCH5B interactions (Chasapis et al., 2012; Birkou et al., 2017), strongly suggesting that UBCH5B utilizes the same interface for its interaction with the Arkadia RING mutant R957C.

The above data show that R957C Arkadia mutant and UBCH5B E2 enzyme interact via the surfaces that are expected to be ivolved in a canonical E2-E3 RING interaction (Chasapis et al., 2012; Gundogdu and Walden, 2019). However, it seems that the CSPs observed for the mutant are somewhat larger compared with those observed for the wt-E2 interaction [0.09/0.06 ppm for the R957C and wt RING, respectively and 0.042/0.038 ppm for the interacting UBCH5B with R957C and wt RING, respectively (Figure 5 and Birkou et al., 2017)].

### Effect of the R957C Mutation on Arkadia’s Function

**FIGURE 7:**
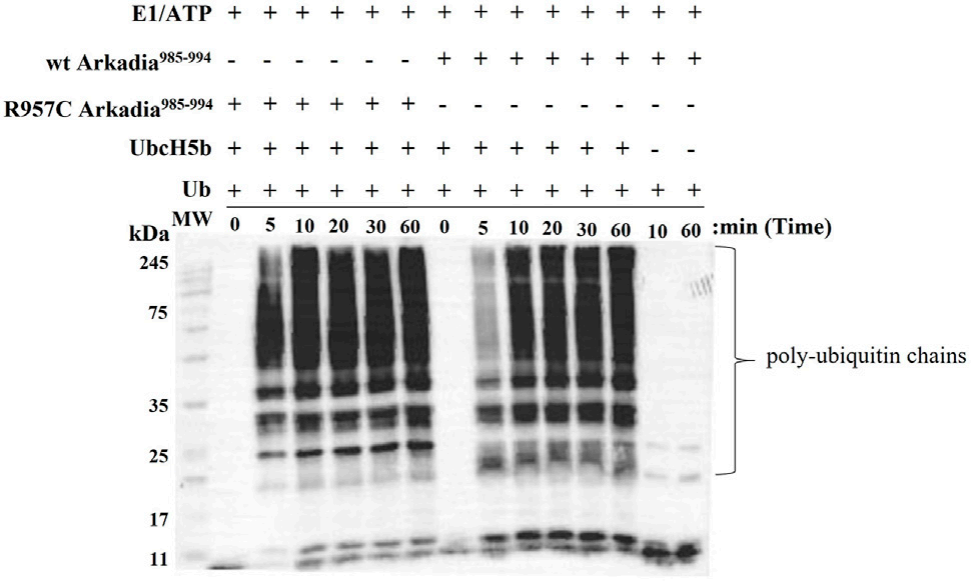
Western blot of *in vitro* ubiquitination assays of R957C Arkadia^985–994^ (left) and wt Arkadia^985–994^ (right).

To examine the actual activity of the mutant protein we developed an *in vitro* ubiquitination assay. For the auto-ubiquitination assays, wt or mutated polypeptides containing NRG and TIER segments along with the RING domain (Arkadia^876–994^) were used, because the RING domain alone does not auto-ubiquitinate. We performed the assay in the presence of E2 UBCH5B and ubiquitin. Immunoblot analysis of these reactions, with anti-ubiquitin antibodies, showed that both wt and R957C Arkadia ^876–994^ were functional in this *in vitro* auto-ubiquitination assay, as indicated from the ladder-like bands that appeared after addition of ATP to the reaction mixture (Figure 7) due to the formation of a polyubiquitin chain on the substrates wt or R957C Arkadia ^876–994^. We found that the R957C Arkadia^876–994^ exhibit significantly rapid and enhanced autoubiquitination (Figure 7), indicating a gain of function and not loss of function.

**FIGURE 8:**
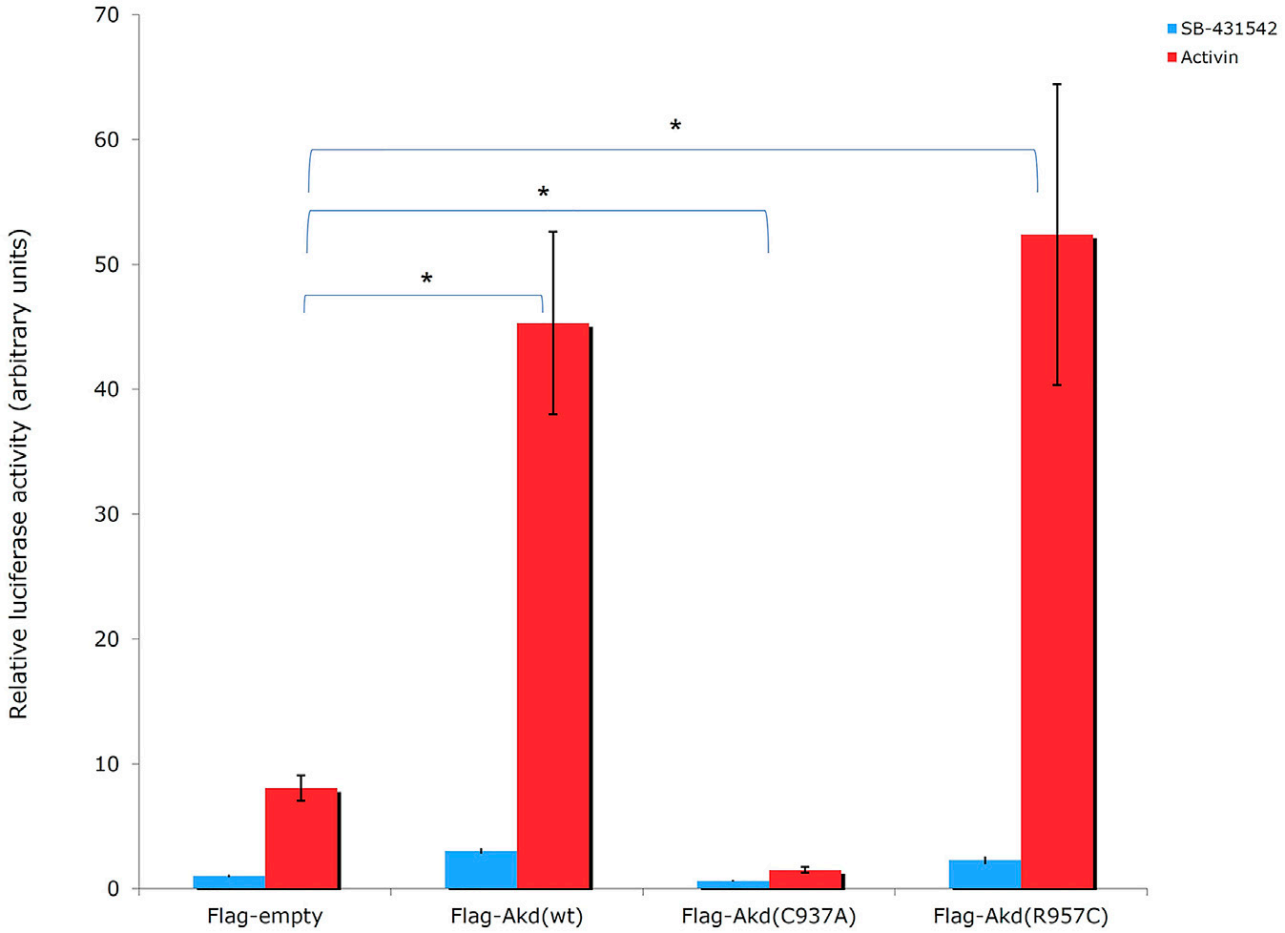
Luciferase CAGA_12_ reporter assay values. Activin: activator of the TGF-*β* pathway. SB-431542: inhibitor of TGF-*β* superfamily type I activin receptor-like kinase receptors. C937A is a ligase defective mutant, which forms mixed dimers (Erker et al., 2013) with the endogenous wt protein leading on suppression of wt function in HEK293T cells (Sharma et al., 2011).

Moreover, R957C Arkadia’s functionality was tested *in vivo* by luciferase assays. Luciferase experiments were carried out in HEK293T cells using the TGF-*β* SMAD-dependent reporter CAGA_12_-Luc, empty plasmid, the full length wt, C937A and R957C Arkadia proteins as described in Birkou et al., 2017. Overexpression of the R957C Arkadia protein enhanced reporter expression a slightly higher than that of the wt Arkadia, however, this was not statistical significant in this assay (Figure 8). These *in vitro* and *in vivo* results show that the R957C mutation on the RING domain does not reduce the activity of Arkadia as E3 ubiquitin ligase.

## Discussion

Mutations within the RING domain of E3 ligases such as BRCA1 are correlated with high risk of cancer (Ruffner et al., 2001). Usually, most of the SNPs cause missense mutations that are neutral or deleterious and unclassified variants. Deleterious SNPs/mutations cause phenotypic differences leading to various types of cancer (Ramensky et al., 2002). Here, we study the structural and functional consequences of the R957C SNP in the RING domain of Arkadia that was identified in normal and tumor tissues of a colorectal cancer patient.

Does the R957C mutation affect the solution structure and dynamics of Arkadia?

**FIGURE 9:**
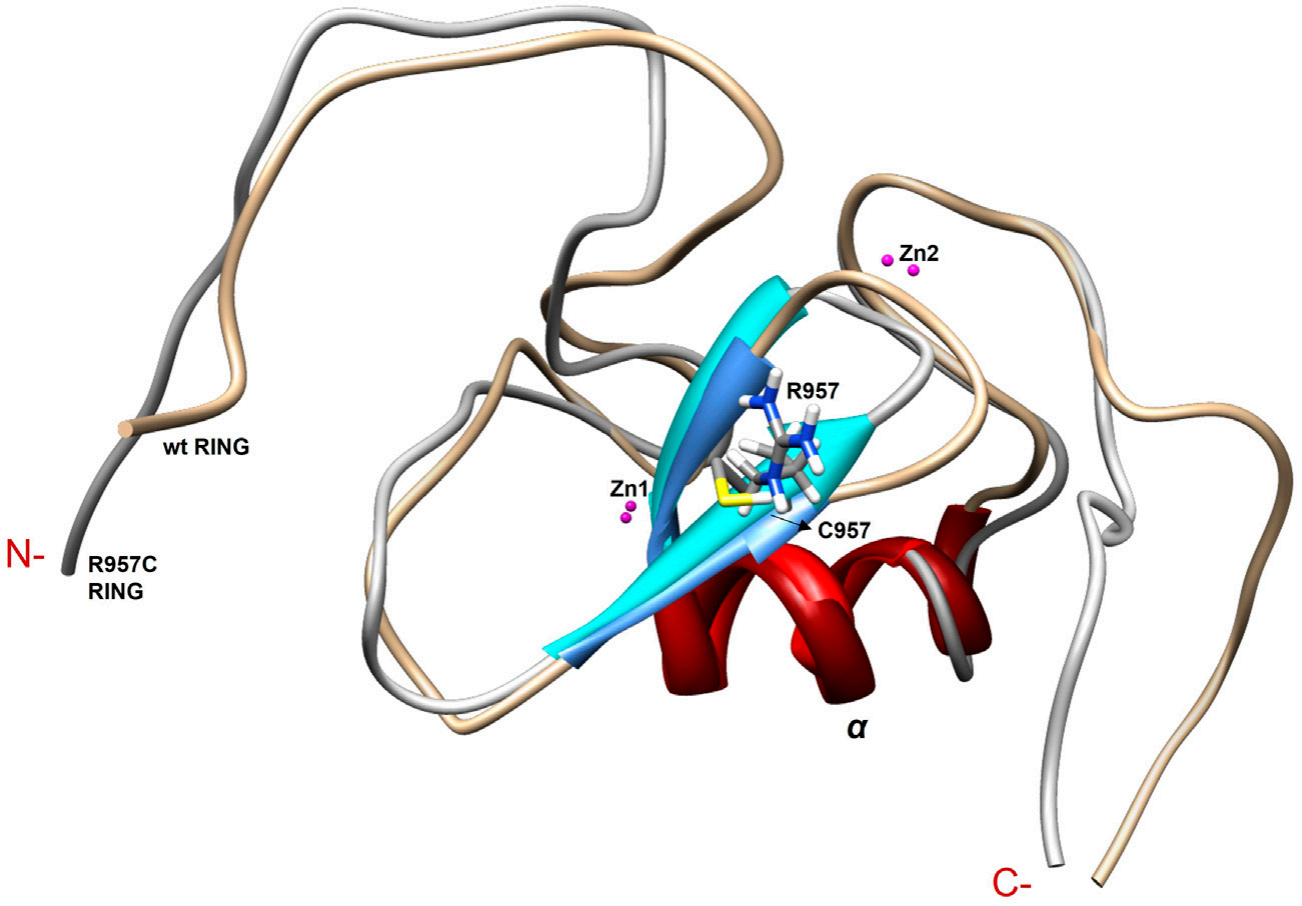
Overlay of the first model of the NMR family models of wt and R957C mutant of Arkadia RING.

**FIGURE 10:**
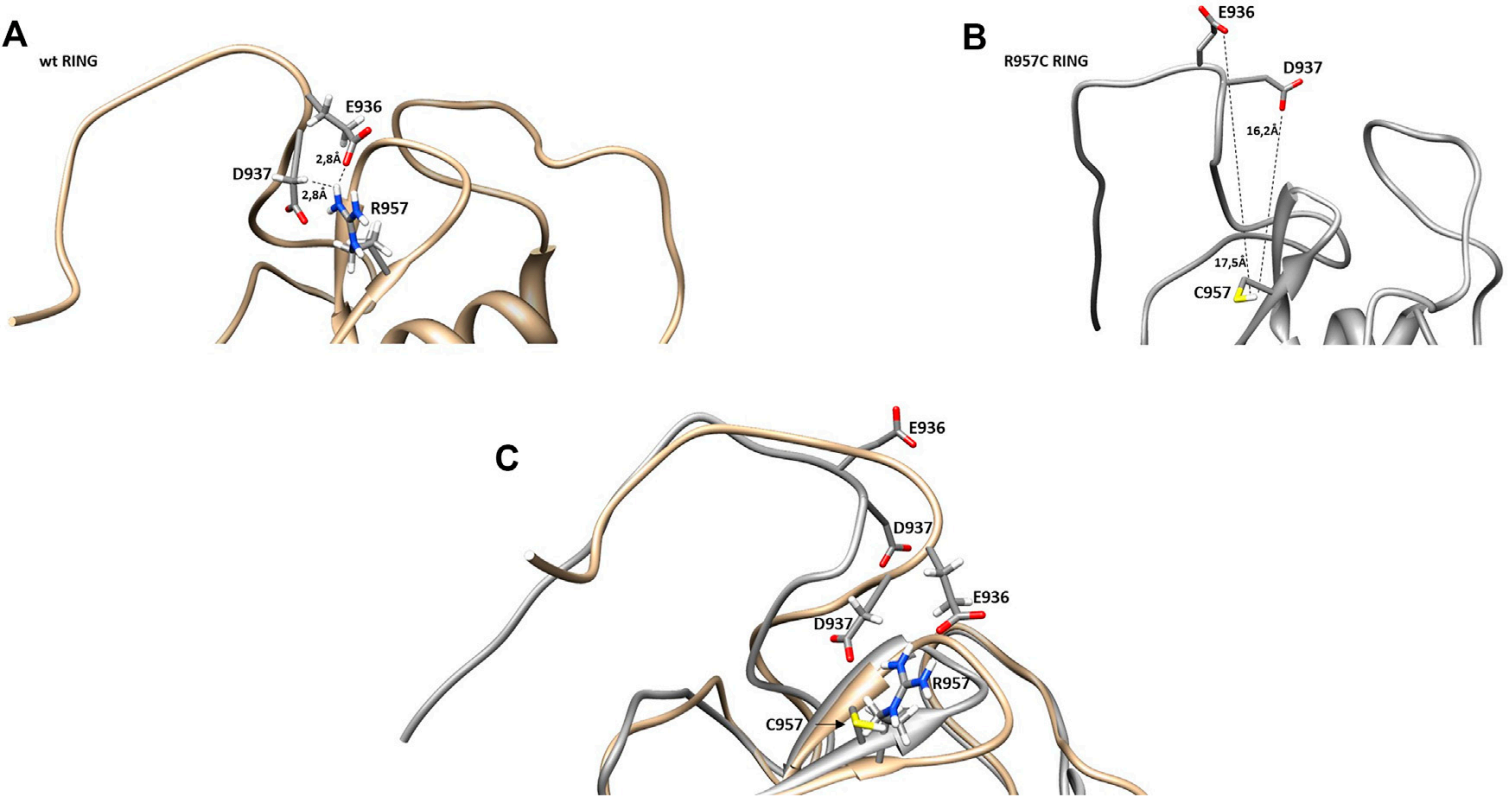
Representation of *β*-sheets region in **(A)** wt and **(B)** R957C RING domain. **(C)** Overlay of the *β*-sheet region of wt (gold) and R957C mutant (white).

The NMR structure of the R957C mutant of Arkadia closely agrees with the NMR structure of the native RING domain (PDB: 2KIZ). Overlay of the first model of R957C RING domain with the first model of wt RING domain shows that there is no significant difference in the overall topology (Figure 9). Both contain a 3-turn *α*-helix, although in the final family of the R957C mutant 10 out of 30 models show a shightly distorted third turn of the *α*-helix due to the lack of helix-diagnostic NOEs. Replacement of Arg957 with Cys results in a slight bending of the *β1*-strand which is slightly bended, affecting the conformation of a loop that comprises the acidic residues Glu936 and Asp937. In wt, the positively charged NH_1_ and NH_2_ atoms of Arg957 interact electrostatically with the carboxyl groups of Glu936 and Asp937, respectively, leading to a compact and less flexible N-terminus (Figure 10). This electrostatic interaction is abolished in the R957C mutant resulting in an increased flexibility of the N-terminus as indicated by the backbone mobility data (Figure 5) and by the distances between the carboxyl groups of Glu936 and Asp937 and the sulfur atom of Cys957. As far as the mobility of the RING core is concerned, the ^15^N relaxation measurements show that the replacement of Arg957 with Cys has essentially no effects on the ps–ns time scale (Supplementary Figure S2).

**FIGURE 11:**
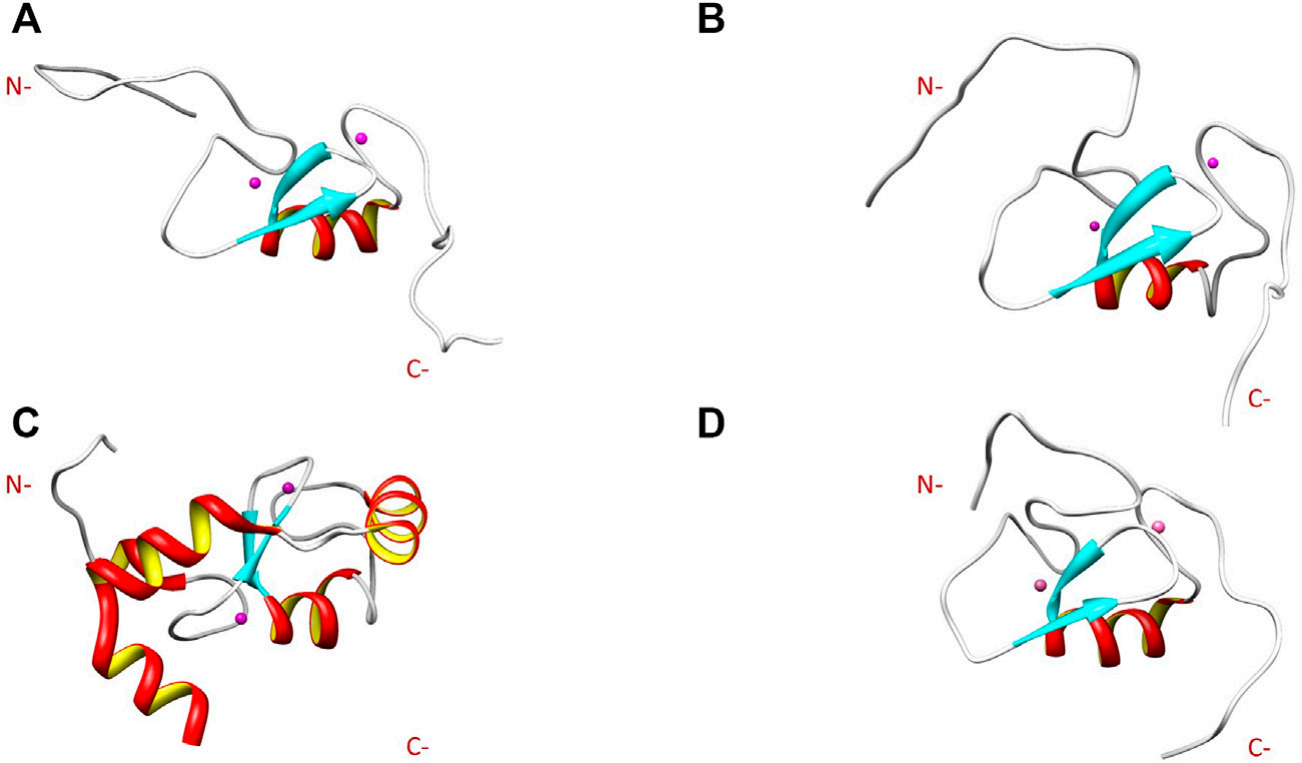
NMR solution structure of **(A)** RNF24 (PDB: 2EP4), **(B)** R957C Arkadia, **(C)** RNF168 (PDB: 4GB0) and **(D)** wt Arkadia (PDB: 2KIZ) RING domains.

**FIGURE 12:**
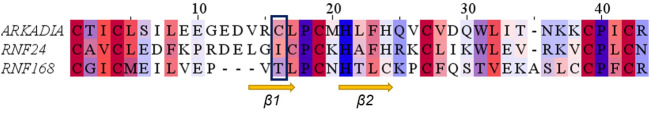
Alignment of the RING domains of R957C Arkadia, RNF24 and RNF168 E3 ubiquitin ligases. Coloring by residue hydrophobicity and sequence conservation.

Comparison of the NMR structure of the R957C mutant with the RING domains of RNF24 (PDB: 2EP4) and RNF168 (PDB: 4GB0) that exhibit a *ββα* topology reveals the similarity of the Arkadia mutant with the RING domain of RNF24 (Figure 11). Interestingly, both domains have *β*-strands, that are four residues long and adopt the same orientation (Figures 11A,B). Moreover, their *β*-strands do not exhibit sequence homology but rather physicochemical similarity in their amino acids composition (Figure 12).

How does the R957C mutation affects Arkadia’s interaction properties and function?

The R957C Arkadia mutant is capable to interact with the E2 enzyme UBCH5B as shown by titration experiments monitored through ^1^H-^15^N HSQC spectra and auto-ubiquitination assays (Figure 6 and Figure 7). Interestingly, the observed CSPs suggest a native-like interaction, involving the two metal-binding loops and parts of the *α*-helix of the mutated RING (Figure 6). A similar surface, formed by the same RING structural elements participates in the E2-E3 interaction of BRCA1 (Brzovik et al., 2003), c-Cbl (Zheng et al., 2000) and RNF38 (Buetow et al., 2015) RING domains. Additionally, the UBCH5B regions that participate in the E2-E3 interaction are the same as those observed in the vast majority of E2-E3 pairs (Gundogdu and Walden, 2019).

**FIGURE 13:**
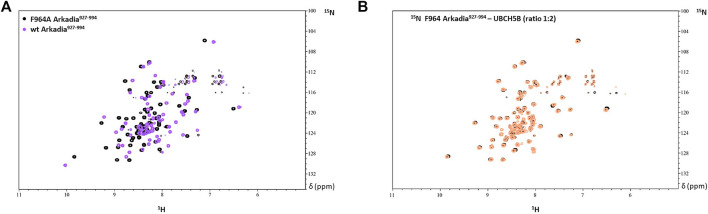
**(A)** Overlay of the ^1^H-^15^N HSQC spectrum of F964A and wt Arkadia RING domain (black and purple, respectively). **(B)** Overlay of the ^1^H-^15^N HSQC spectrum of F964A before (black) and after (orange) the addition of UBCH5B to a 1:2 ratio.

Thus, the R957 mutation which is located on the *β1*-strand, does not seem to have an impact on the RING domain interaction properties. Since R957 is not considered as a residue that is crucial for E2-E3 interaction (Chasapis et al., 2012) its mutation to cysteine is not expected to disrupt the interaction with the E2 enzyme UBCH5B. Generally, it is well established that mutations of conserved residues in RING domains disrupt E2 interaction and result in the loss of E3 ligase enzymatic activity (Deshaies and Joazeiro et al., 2009). For example, mutation of the conserved tryptophan (Trp) in the *α*-helix of most RING domains disrupts the recruitment of E2 and this is sufficient to abolish the ligase activity. A recent study on Arkadia’s RING domain revealed that mutation of Trp972 to arginine affected Arkadia’s interaction interface and abolished the E3 ligase function (Birkou et al., 2017). Another example is the mutation of Phe964 in the Arkadia RING domain. This phenylalanine is conserved in RING-H2 ligases and precedes the fifth zinc binding residue. Mutation of F964 to alanine (Ala, A) changed the folding of the RING domain (Figure 13A), which resulted in the disruption of E2-E3 interaction (Figure 13B).

The auto-ubiquitination assay revealed R957C mutation is a gain of function mutation that enhances the enzymatic activity of the RING (Figure 7), and consistent to this, the luciferase assays shows that the R957C mutation exhibits slightly increased activity of Arkadia although it is not statistically significant here (Figure 8). The TGF-*β* pathway has tumor suppressive properties in normal tissues and promotes metastasis within tumors. Similarly, Arkadia enhances TGF-*β* signaling and exhibit the same bidiretional properties (Sharma et al., 2011; Briones-Orta et al., 2013). Indeed, Sharma et al., 2011 has shown that reduction of Arkadia in normal cells increases significantly the susceptibility to cancer. Furthermore, Le Scolan et al., 2008 and Briones-Orta et al., 2013 performing loss of function experiments in tumor cell lines have shown that Arkadia supports metastatic phenotypes via a TGF-*β* dependent manner. Our data presented here support the pro-tumorigenic functions of Arkadia in a context-cell type-dependent manner because it was found enriched in tumor compared to the adjacent normal tissue. However, it is possible that the presence of the R957C mutation in the adjacent normal cells represent infiltration of a metastatic tumor cells and not a polymorphism. More experiments are necessary to verify the role of this hyperactive mutant Arkadia within tumor cells.

## Conclusion

Analysis of the consequences of SNPs on the 3D protein structure is beneficial in understanding both their function and their role in diseases. SNPs at the level of proteins usually affect their activity, binding/association, assembly, and rearrangement and promote their aggregation (Bhattacharya et al., 2017). Most studies analyzing the effects of SNPs on the 3D protein structure are restricted to specific diseases. The goal of this study is to provide experimental data about an SNP that was found in CRC and adjacent normal tissue samples of a patient and its effects on structure, stability, binding properties, and function of the Arkadia RING domain. Our NMR structural data show that the R957C mutation, in the *β1* strand of this domain is located far away from the two Zn (II) binding motifs. It does not interfere with the metal binding and does not alter the geometry of the cross-brace metal coordination; moreover, it does not change the conformation of the E2 docking surface of the RING domain. The R957C mutation does not lead to conformational change in the *β1−β2* surface, so no significant change of the overall conformational features of the RING domain is observed, and the same holds for the backbone dynamics of the RING core. Finally, the functional assays (Figures 7, 8) also showed that Arkadia does not lose its E3 ligase activity, despite the R957C mutation. It is worth mentioning that auto-ubiquitination assays indicated a gain of function effect of the R957C mutation of Arkadia, while the luciferase functional assay showed milder hyperactivity. The finding that this SNP was present in normal tissue and enriched within the tumor, suggests that it segrates with the tumor and possibly with infiltration of adjacent tissue by metastatic tumor cells.

## Data Availability

The datasets presented in this study can be found in online repositories. The names of the repository/repositories and accession number(s) can be found in the article/Supplementary Material.
