# Supplementary material for: Impact of a Single Nucleotide Polymorphism on the 3D Protein Structure and Ubiquitination Activity of E3 Ubiquitin Ligase Arkadia
Source: Front Mol Biosci. 2022 Feb 23;9:844129. doi: 10.3389/fmolb.2022.844129 (PMC8905620; doi:10.3389/fmolb.2022.844129)
Supplement: Supplementary file 1 [file DataSheet1.docx]

Supplementary Material

**Supplementary Tables**

**Table S1:** Representation of the case number corresponding to the R957C (CGT>TGT) mutation and frequency of the mutation identified in cancer and normal tissues by PCR using 5΄-tttagaggaaggtgaagatgtgag-3΄and 5΄-aaccaatggttattcttcacg-3΄ pair or primers.

| **Case Number**  **(Pathology**  **Report)** | **Type of tissue** | **Total No. Of Clones screened (PCR region)** | **Mutation identified in PCR region (frequency %)** |
| --- | --- | --- | --- |
| 11056 | ca | 125 | 48 |
| - | normal | 47 | 17 |

**Table S2:** Statistical analysis of the ensemble of 30 energy minimized models of R957C Arkadia.

|  | **Arkadia R957C**  **(30 Conformers) ^a^** |
| --- | --- |
| Total number of meaningful NOE upper distance constraints | 733 |
| Intra-protein NOEs ^c^ | |
| Intra-residue | 205 |
| Inter-residue | 528 |
|  |  |
| **RMS violations per meaningful distance (Å)** | |
| Intraresidue | 0.025 ± 0.003 |
| Sequential | 0.021 ± 0.003 |
| Medium range | 0.012 ± 0.004 |
| Long range | 0.026 ± 0.004 |
| AVERAGE VALUES | 0.022 ± 0.002 |
| Phi angles | 0.594 ± 0.557 |
|  |  |
| **Average number of violations per conformer** | |
| Intraresidue | 11.8 ± 2.30 |
| Sequential | 12.17 ± 2.41 |
| Medium range | 3.53 ± 1.38 |
| Long range | 7.23 ± 1.54 |
| TOTAL | 34.73 ± 3.51 |
| Phi angles | 0.63 ± 0.61 |
|  |  |
| NOE violations larger than 0.3 Å | 1 |
|  |  |
| **Average RMSD (Å)** | |
| Residue range 937-988 (backbone atoms) | 1.22 ± 0.48 |
| Residue range 937-988 (all heavy atoms) | 2.08 ± 0.45 |
|  |  |
| **Structural analysis ^d^** | |
| % of residues in most favorable regions | 56.0 |
| % of residues in allowed regions | 33.3 |
| % of residues in generously allowed regions | 8.3 |
| % of residues in disallowed regions | 2.4 |
|  |  |
| AMBER energy (kJ mol^-1^) | -2195.10 ± 202.29 |
|  |  |
| ^a^ Structure calculations were performed with the program DYANA(1). Each member of the family was subjected to restrained energy minimization (REM) with the AMBER 5.0 package (2). Values of 32 kcal mol^-1^ Å^-2^ and 32 kcal mol^-1^ rad^-2^ were used as force constants for the NOE and torsion angle restraints, respectively. The data are calculated over the 30 conformers representing the NMR structure and on the energy minimized mean structure. The mean value and the standard deviation are given.  ^b^ Average model derived from the ensemble of 30 models and then subjected to restrained energy minimization as described above for the 30 conformers  ^c^ Number of meaningful constraints for each class.  ^d^ As it results from the Ramachandran plot analysis performed with PROCHECK (3). | |

**Table S3**: Acquisition parameters of the NMR experiments performed on R957C Arkadia at 600 MHz and 700 MHz (298 K).

| Experiments^a^ | Dimension of acquired data  (nucleus) | | Spectral width  (ppm) | | n^b^ |
| --- | --- | --- | --- | --- | --- |
|  | t_1_ t_2_ | | F_1_ F_2_ | |  |
| [^1^H-^15^N]-HSQC | 256(^15^N) | 2048(^1^H) | 26 | 17.9 | 12 |
| ^15^N R_1_ | 512(^15^N) | 2048(^1^H) | 26 | 15 | 24 |
| ^15^N R_2_ | 512(^15^N) | 2048(^1^H) | 26 | 15 | 24 |
| steady-state heteronuclear NOEs | 512(^15^N) | 1024(^1^H) | 26 | 15 | 56 |
| ^a^ ^15^N *R*1 and *R*2 relaxation rates (4) and {^1^H^N^}–^15^N NOEs values (5) were acquired at 600 MHz and 700 MHz spectrometers equipped with a 5 mm triple resonance cryoprobe (TXI and TCI, respectively) with pulsed field gradients along the z-axis.  ^b^ number of acquired scans.  All 2D and 3D spectra were collected at 298 K, processed with the Bruker software TOPSPIN and analyzed by the programs CARA (6). | | | | | |

**Table S4:** The distances of NH_1_ and NH_2_ of Arg957 and S_γ_ of C957 to O_δ1_,Ο_δ2_ and Ο_ε1_, Ο_ε2_ of D937 and E936, respectively, as measured from the 30 final models of wt and R957C polypeptide.

|  | | **R957** | | **C957** |
| --- | --- | --- | --- | --- |
|  |  | NH_1_ | NH_2_ | S_γ_ |
| **D937** | O_δ1_ | 5.88±2.09 (min=2.78Å) | - | 13.25±2.70 (min=7.52Å) |
|  | Ο_δ2_ | 5.04±2.21 (min=2.70Å) | - | 13.11±2.90 (min=5.51Å) |
| **E936** | Ο_ε1_ | - | 8.17±3.85 (min=2.81Å) | 13.77±3.6 (min=4.02Å) |
|  | Ο_ε2_ | - | 7.35±4.38 (min=2.77Å) | 13.63±3.17 (min=5.71Å) |

**Supplementary Figures**

**Figure S1:** Calibration curve for zinc standard solutions absorption.


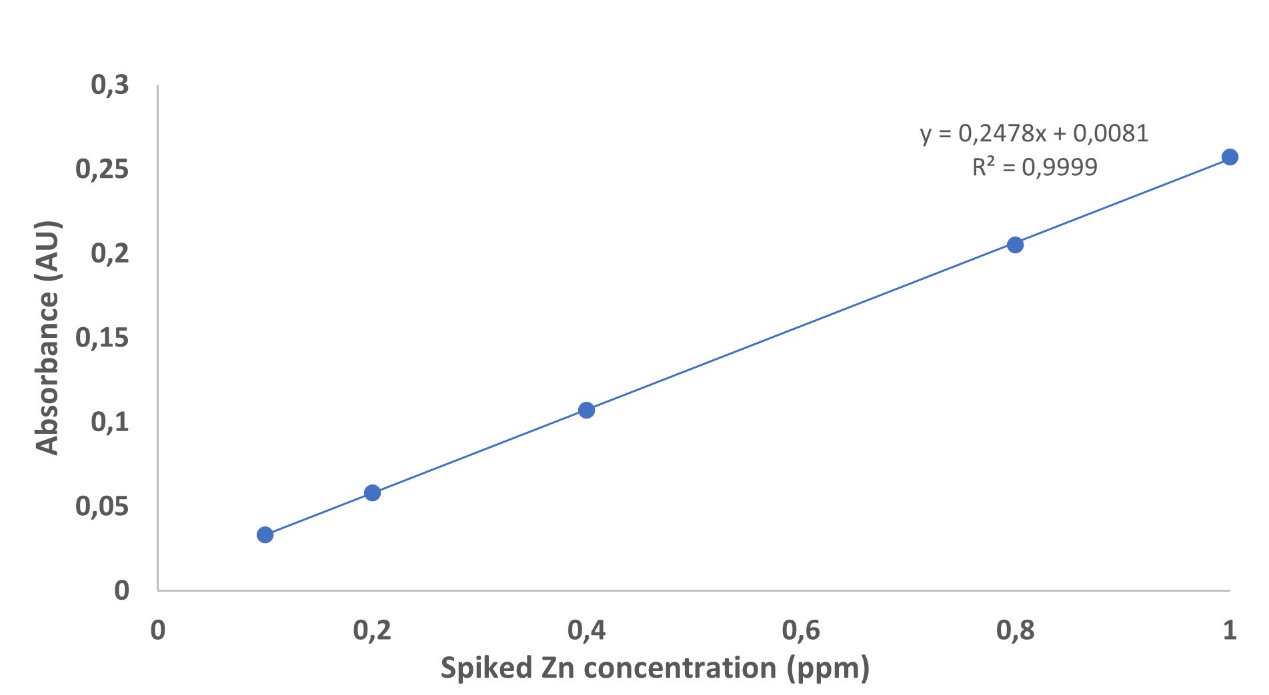


**Figure S2:** ^15^N-relaxation data (*R*_1_), (*R*_2_) and {^1^H}-^15^N NOE of R957C Arkadia mutant collected at 600MHz and 298 K.


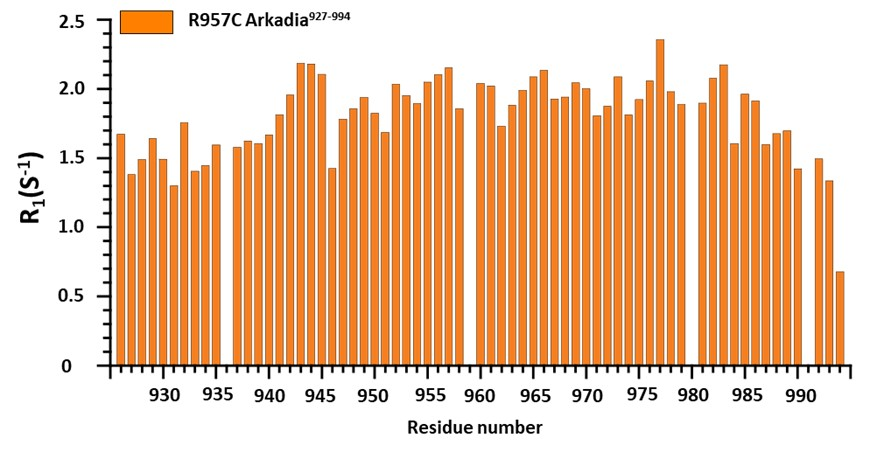


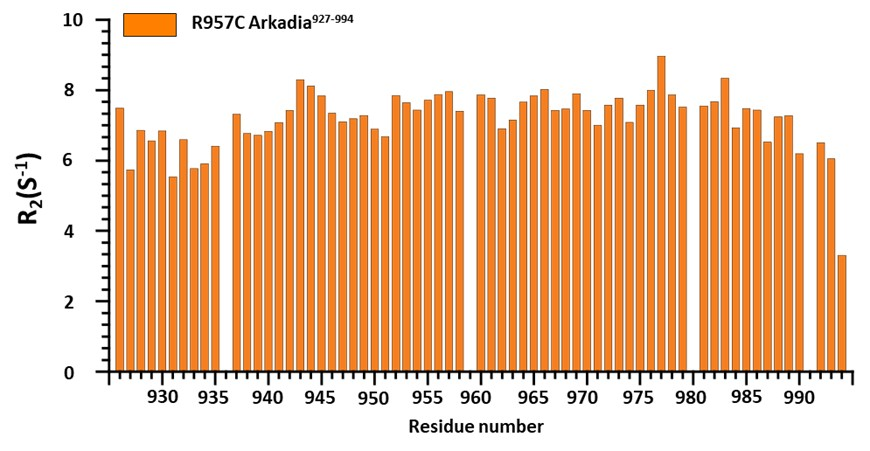


**
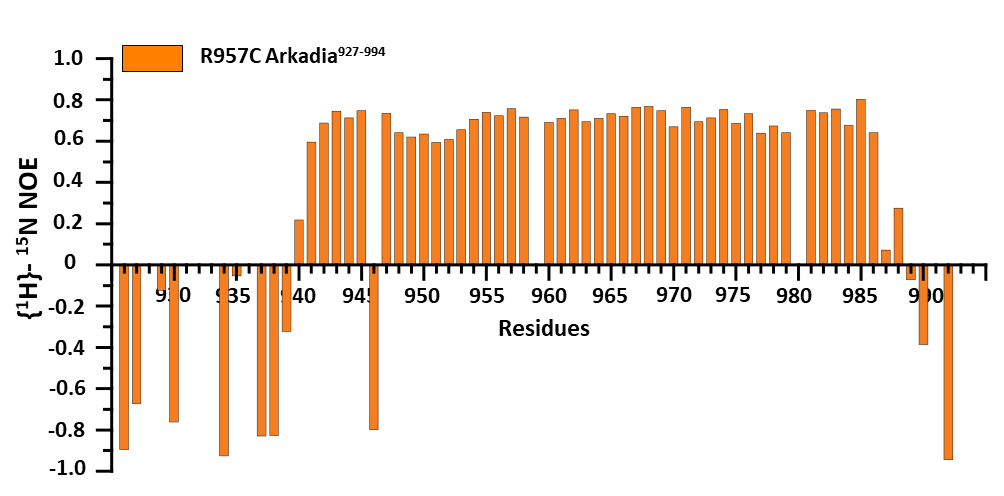
**

**REFERENCES**

1. Güntert, P., Mumenthaler, C., Wüthrich, K. (1997). J. Mol. Biol. 273, 283-298. doi:10.1006/jmbi.1997.1284.

2. Pearlman, D.A., Case, D.A., Caldwellm, J.W., Ross, W.S., Cheatham, T.E., Ferguson, D.M., Seibel, G.L., Singh, U.C., Weiner, P.K., Kollman, P.A. (1997). AMBER 5.0, University of California, San Francisco.

3. Laskowski, R.A., MacArthur, M.W., Moss, D.S., Thornton, J.M. (1993). J. Appl. Crystallogr. 26, 283–291. [doi.org/10.1107/S0021889892009944](https://doi.org/10.1107/S0021889892009944).

4. Farrow, N.A., Muhandiram, R., Singer, A.U., Pascal, S.M., Kay, C.M., Gish, G., Shoelson, S.E., Pawson, T., Forman-Kay, J.D., Kay, L.E. (1994). Biochemistry 33, 5984–6003. doi: 10.1021/bi00185a040.

5. Grzesiek, S., Bax, A. (1993). J. Am. Chem. Soc. 115, 12593–12594.

6. Keller R. 2004, Cantina Verlag, CH-6410 Goldau.
